# Continuous Theta-Burst Stimulation Demonstrates a Causal Role of Premotor Homunculus in Action Understanding

**DOI:** 10.1177/0956797613520608

**Published:** 2014-04

**Authors:** John Michael, Kristian Sandberg, Joshua Skewes, Thomas Wolf, Jakob Blicher, Morten Overgaard, Chris D. Frith

**Affiliations:** 1 Center for Subjectivity Research, Copenhagen University; 2 Interacting Minds Centre, Aarhus University; 3 Cognitive Neuroscience Research Unit, Hammel Neurorehabilitation and Research Centre, Aarhus University; 4 Institute of Cognitive Neuroscience, University College London; 5 Program in Cognitive Science, University of Vienna; 6 Cognitive Neuroscience Research Unit, Center of Functionally Integrative Neuroscience/MindLab, Aarhus University; 7 Centre for Cognitive Neuroscience, Department of Communication and Psychology, Aalborg University; 8 Wellcome Trust Centre for Neuroimaging, London, England

**Keywords:** mirror-neuron system, action understanding, theta-burst stimulation, social cognition, theory of mind, social interaction, social perception

## Abstract

Although it is well established that regions of premotor cortex (PMC) are active during action observation, it remains controversial whether they play a causal role in action understanding. In the experiment reported here, we used off-line continuous theta-burst stimulation (cTBS) to investigate this question. Participants received cTBS over the hand and lip areas of left PMC, in separate sessions, before completing a pantomime-recognition task in which half of the trials contained pantomimed hand actions, and half contained pantomimed mouth actions. The results reveal a double dissociation: Participants were less accurate in recognizing pantomimed hand actions after receiving cTBS over the hand area than over the lip area and less accurate in recognizing pantomimed mouth actions after receiving cTBS over the lip area than over the hand area. This finding constrains theories of action understanding by showing that somatotopically organized regions of PMC contribute causally to action understanding and, thus, that the mechanisms underpinning action understanding and action performance overlap.

Mirror neurons, originally discovered in macaque monkeys using single-cell recordings, are active when an animal is either performing a particular action or observing another agent performing the same or a similar action (di Pelligrino, Fadiga, Fogassi, Gallese, & Rizzolatti, 1992; Gallese, Fadiga, Fogassi, & Rizzolatti, 1996; Rizzolatti, Fadiga, Gallese, & Fogassi, 1996). Because single-cell activity has rarely been recorded in humans (but see Mukamel, Ekstrom, Kaplan, Iacoboni, & Fried, 2010), most research involving human participants has been performed with imaging techniques that measure activity in brain regions rather than in individual cells. This research has revealed a network, comprising regions of premotor cortex (PMC), inferior parietal lobule, and somatosensory areas (Buccino et al., 2001; Gazzola & Keysers, 2009; Iacoboni et al., 2005; Rizzolatti & Craighero, 2004; Vogt et al., 2007), that is “activated during performance of [an] action as well as during the observation [of] the same action being performed by another person” (Frith & Singer, 2008, p. 3876). On the basis of these findings, it has been proposed that this network, sometimes called the *mirror-neuron system* (MNS), plays a causal role in action understanding (i.e., in identifying the goals, or underlying intentions, of bodily movements) and that action production and action understanding involve overlapping mechanisms (Gallese, Keysers, & Rizzolatti, 2004; Gazzola & Keysers, 2009; Iacoboni et al., 2005; Kilner, Neal, Weiskopf, Friston, & Frith, 2009; Pobric & Hamilton, 2006; Vogt et al., 2007). However, because most of the research in this area has relied on correlational methods, these claims have remained controversial, with competing models offering conflicting accounts of the function of MNS activation.

According to the *direct-matching* model, activation of the PMC during action observation constitutes a covert simulation of the observed action, which enables the observer to match it with an action in his or her own repertoire of intentional actions and thereby to identify the goal of the action (Gallese et al., 2004). The direct-matching model therefore holds that somatotopically organized regions of PMC play a causal role in understanding observed actions. The *predictive-coding* model (Kilner, Friston, & Frith, 2007) is based on the conception of a hierarchy of reciprocally connected models. Each model generates predictions about the representations at the immediately subordinate level. These predictions are compared with the actual state of the subordinate-level model, and a prediction error is returned to the superordinate-level model, which is revised and then generates a new prediction. By this process, the interconnected models are continuously updated and prediction errors minimized. Thus, according to the predictive-coding model, premotor activation and higher-level representations reciprocally modulate each other. Like the direct-matching model, then, the predictive-coding model holds that somatotopically organized regions of PMC play a causal role in action understanding, with the mechanisms for action understanding overlapping with those for the production of actions.

In contrast, two deflationary models deny that premotor activation plays a causal role in action understanding. According to what can be called the *priming model*, putative mirroring properties may in fact support sensory-motor associations, in which case premotor activation during action observation might reflect a priming effect rather than a contribution to action understanding (Hickok, 2009). According to what can be called the *inverse-modeling* model, the function of premotor activation during action observation is to calculate motor commands appropriate to the realization of a goal and thus to predict the upcoming movements, given that the goal has already been identified by other means (Csibra, 2008). In other words, premotor activation, according to this model, is a result rather than a cause or component of action understanding.

In the experiment reported here, we used continuous theta-burst stimulation (cTBS), an off-line protocol for transcranial magnetic stimulation (TMS), to investigate whether PMC plays a causal role in action understanding and thus to adjudicate between models that affirm this (e.g., direct matching and predictive coding) and models that deny it (e.g., priming and inverse modeling). It has been documented that the application of cTBS over motor areas diminishes the excitability of cortical tissue for approximately 20 min (Huang, Edwards, Rounis, Bhatia, & Rothwell, 2005; Huang et al., 2009). We therefore administered cTBS over participants’ premotor hand and lip areas, in separate sessions, and then measured their performance on a series of tasks designed to probe the mechanisms underlying action understanding. We reasoned that if premotor hand and lip areas play a causal role in action understanding, then the application of cTBS over the premotor hand area should specifically impair participants’ ability to process observed hand actions, and the application of cTBS over the premotor lip area should specifically impair their ability to process observed mouth actions.

Because identifying the goals of bodily movements is a complex process that likely involves multiple components, we agree with Cook, Bird, Catmur, Press, and Heyes (in press) that research on the contribution of the MNS to action understanding must attempt to isolate and operationalize distinct components of action understanding. Drawing on a tripartite hierarchical distinction developed by Hamilton and Grafton (2007), we therefore designed three separate tasks of varying complexity in order to probe different components of action understanding. The simplest task required participants to identify still frames from brief videos of pantomimed actions. This task thus probed a perceptual aspect of action understanding, namely the ability to process kinematic features of observed actions. An intermediate task required them to select which of three objects complemented a brief video of a pantomimed action, thus probing their ability to identify the proximal goal of an observed movement. The most complex task required them to select which of three objects complemented a brief video of a pantomimed action in a context-sensitive manner, thus probing their ability to identify the distal goal of an observed movement (see Fig. 1 and Table 1). Whereas the simple task did not require participants to identify the goal of the observed action, it is an open question whether the processing of low-level kinematic features of actions plays a role in the identification of goals. The intermediate and complex tasks pertain directly to action understanding insofar as they required participants not just to process perceptual features of bodily movements but also to identify the goals, or underlying intentions, of those movements.

**Fig. 1.**
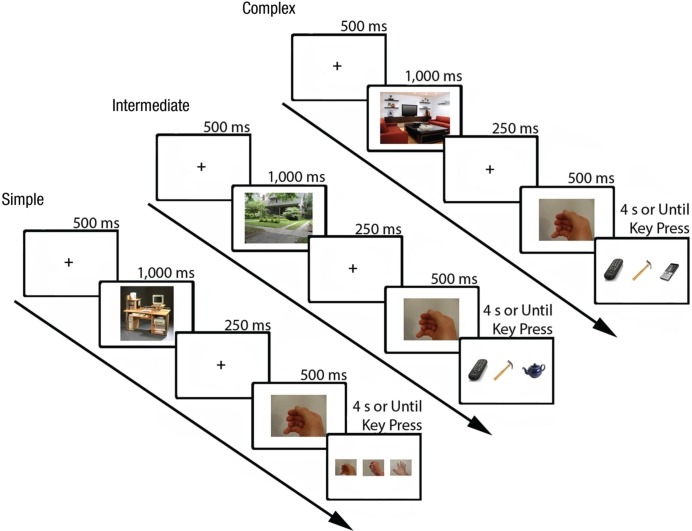
Example trial sequence for each complexity level. On each trial, an image depicting the context for a subsequent video was displayed for 1,000 ms, followed by a black fixation cross, then the video of a pantomimed action. Participants next saw a response screen, on which three static images were displayed for 4 s or until a response was given. In the simple block, participants chose which one of the three images was a still frame from the action video they had just seen. In the intermediate block, participants were asked to choose the object that best complemented the action pantomimed. The complex block differed from the intermediate block only in that two of the objects on the response screen complemented the action video but only one complemented the context, thus making it necessary for participants to draw on contextual information as well as to observe the pantomimed action in order to choose the correct object. In all three examples depicted here, the correct response was the image on the left.

**Table 1.** Example Stimuli for Hand- and Mouth-Action Trials in the Intermediate and Complex Blocks

Action video and block	Context image	Response options
Hand action		
Turning		
Intermediate	House entrance	Newspaper, **key**, envelope,
Complex	Workbench	*Key*, hammer, **screwdriver**
Pouring		
Intermediate	Tea party	**Teapot**, sugar bowl, spoon
Complex	Plants in garden	**Watering can**, hedge clippers, *teapot*

Mouth action		
Licking		
Intermediate	Beach	**Ice cream cone**, soda can, burger
Complex	Desk with papers	*Ice cream cone*, eraser, **stamp**
Blowing		
Intermediate	Birthday party	Cake on fork, **candles on cake**, presents
Complex	Romantic dinner	Wine glass, **one candle**, *candles on cake*

Note: On all trials, participants saw an image that provided context for a subsequent video, followed by the video itself; the video depicted either a hand or a mouth action. Participants were then shown three new images, from which they had to choose the one that best complemented the video. The correct response is shown here in boldface. For the intermediate trials, only one of the response options fit the action video, thus rendering the context image superfluous. For the complex trials, a second response option (shown here in italics) fit the action video but not the context, whereas the third response option fit the context but not the action, thus making it necessary to integrate the context image and the action video in order to choose correctly.

## Method

### Participants

Twenty right-handed individuals (8 females, 12 males; mean age = 23.5 years, range = 18–40) took part in the experiment. All were naive to the experiment’s purpose and gave their informed written consent. They had no history of epilepsy and were not taking any medication at the time of the test. One further participant, a 20-year-old male, experienced a vasovagal episode (unrelated to the stimulation) during the localization procedure prior to the cTBS and therefore discontinued his participation.

### Procedure

**Fig. 2.**
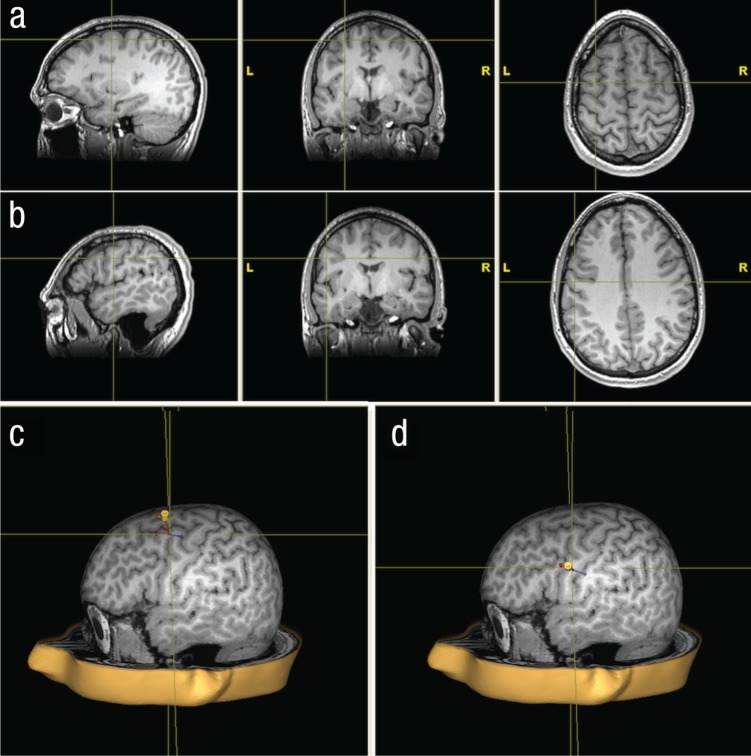
Stimulation sites for 1 participant. The crosshairs in (a) and (b) mark the hand area and lip area, respectively, in premotor cortex (PMC) that were stimulated in this participant. The images were used to create a 3-D brain model, depicted in the bottom row, to be utilized in conjunction with the navigation system. In (c), the hand area is at the crosshair, and in (d), the lip area is at the crosshair. The marker illustrates the position and orientation of the coil during cTBS. L = left, R = right.

Each participant took part in a preliminary session in which a T1 structural scan was performed, and then in two experimental sessions, each of which was preceded by a TMS session (see Fig. 2). Each TMS session consisted of a localization procedure followed by the administering of cTBS. In one session, cTBS was applied over the hand area in left PMC, whereas in the other session, it was applied over the lip area in left PMC. The order of the sessions was counterbalanced. After completing their second experimental session, participants were debriefed. The ethics committee for the region of Midtjylland, Denmark, approved the experiment.

Participants first completed a practice block of 50 trials, which were not repeated in the experiment. Then they completed three separate test blocks, which corresponded to three levels of complexity (see Fig. 1) and which were presented in counterbalanced order. In each block, participants completed 80 trials (depicting 40 hand actions and 40 mouth actions), the order of which was randomized. Participants were instructed to complete each trial as quickly and accurately as possible.

In all blocks, each trial began with a 500-ms black fixation cross. Then an image depicting a context for the video they were about to see was displayed for 1,000 ms. The context image was followed by a second fixation cross (250 ms), then a video of a pantomimed action (500 ms). Participants next saw a response screen on which three static images were displayed from left to right across the middle of the screen for 4 s or until a response was given. The task differed slightly across the three blocks. In the simple block, the three images were of a hand or mouth, and participants were told to “choose the image that depicts the same action as the video you just saw.” In the intermediate block, participants saw different objects and were asked to “choose the object that fits best with the action and the setting you just saw.” The complex block differed from the intermediate block only in that two of the objects on the response screen fit the action video, but only one fit the context, thus making it necessary for participants to draw on contextual information as well as observe the pantomimed action in order to choose the correct object. For all three blocks, the response was given by pressing the 1, 2, or 3 key with the first three fingers of the left hand.

### Stimuli

The stimuli were brief videos of pantomimed mouth actions (e.g., licking, sucking, blowing) and hand actions (e.g., writing, cutting, grasping). We used 20 different mouth-action videos and 20 different hand-action videos (see Table 1 for examples), each being repeated six times per experimental session (twice per block). There were 60 context images, each of which was repeated four times per experimental session. There were 120 images used as response options, each of which was repeated six times. The stimuli were presented using E-Prime software (Schneider, Eschman, & Zuccolotto, 2001) and were displayed on a 13-in. LCD monitor (refresh rate: 60 Hz, resolution: 1,280 × 800 pixels).

### Data acquisition

A T1-weighted magnetic resonance imaging volume was acquired for each subject with a GE Signa Excite HDx 3.0-T spectrometer using a 3-D inversion–recovery prepared fast spoiled-gradient-recalled sequence (echo time = 3.0 ms, inversion time = 450 ms, flip angle = 20°, slices = 156, slice thickness = 1.1 mm, in-plane resolution = 0.94 mm).

The scans were used in conjunction with a Nexstim (Helsinki, Finland) navigator device and NBS 3.2 software during the localization procedure and during the cTBS in order to record the site and orientation of the coil as each pulse was administered, as well as the intensity of each pulse and the elicited motor-evoked potential (MEP). A Magstim X100 stimulator (MagVenture, Farum, Denmark) was used with an MCF-B65 figure-of-eight coil (MagVenture) to generate the cTBS; 300 pulses were administered in 100 bursts of 3 pulses each over a 20-s period. The frequency within each burst was 50 Hz, and the bursts were repeated with a frequency of 5 Hz. Stimulation during cTBS was given at 70% of the resting motor threshold (RMT), based on previous studies reporting that 70% of the RMT is equivalent to 80% of the active motor threshold (Chen et al., 1998; Gentner, Wankerl, Reinsberger, Zeller, & Classen, 2008).

It has been shown that cTBS over motor areas inhibits subsequent MEPs only if preceded by a period of slight voluntary contraction lasting at least 1.5 min and that it otherwise facilitates MEPs (Gentner et al., 2008; Huang et al., 2009). For this reason, participants were asked immediately prior to the administering of cTBS in this experiment to clench their fist while relaxing their lip, and then to relax their fist while squeezing their lips together, in alternation, for 5 min.

### Localization

For the localization procedure, the hand area in left primary motor cortex (M1) was first approximated by sight with the help of the navigation system. Then, slightly suprathreshold pulses were administered over 10 nearby sites, and the optimal site was defined as the location where stimulation elicited largest MEPs in the first dorsal interosseous muscle of the right hand. The RMT was determined as the lowest intensity at which MEPs above 50 µV were recorded on 5 of 10 trials. For the hand area, the mean threshold intensity was 42% of maximum stimulator output. The procedure was repeated for the lip area, with MEPs being recorded on the orbicularis oris, starting from a site 3 cm lateral and 1.5 cm anterior to the hand site. The mean RMT for the lip area was 56%. The premotor sites were then determined by moving 3 cm anterior from the corresponding spots in M1 and then to the nearest spot on the precentral gyrus with the help of the navigation system.

Whereas prior TMS studies have localized the hand area in left PMC using the same method as that employed here (Kroeger et al., 2010; Münchau, Bloem, Irlbacher, Trimble, & Rothwell, 2002), there is no precedent for the use of the same localization procedure for the premotor lip area. However, the extension of this procedure to the lip area derives support from several sources. First, studies with nonhuman primates suggest a somatotopic organization of PMC that parallels that in M1, with higher concentrations of neurons encoding features of leg and foot movements in more dorsal areas than hand representations and lip representations in more ventral areas (Gentilucci et al., 1988; Godschalk, Mitz, van Duin, & van der Burg, 1995; Rizzolatti et al., 1988). Second, imaging studies in humans have corroborated this pattern (Buccino et al., 2001; Hauk, Johnsrude, & Pulvermüller, 2004; for a meta-analysis, see Schubotz & von Cramon, 2003). Third, lesion studies have confirmed that patients with lesions in more dorsal areas of PMC have greater difficulties performing hand actions, whereas patients with more ventral lesions have greater difficulties performing mouth actions (Basso, Capitani, Della Sala, Laiacona, & Spinnler, 1987; Marquart & Sussman, 1984; Pazzaglia, Smania, Corato, & Aglioti, 2008). Thus, although it is not likely that the regions of PMC that we localized are uniquely specialized for hand and mouth actions, respectively, there are independent reasons to accept that the area we defined as the premotor lip area contains neural populations that specialize in the production of mouth actions and that the area we defined as the premotor hand area contains neural populations that specialize in hand-action production.

Because the lip area served as an optimal control site with respect to the effects of cTBS over the hand area on hand-action understanding, and the hand area in turn served as a control site with respect to the effects of cTBS over the lip area on mouth-action understanding, it was not necessary to include an additional control site or a sham TMS condition.

## Results

**Fig. 3.**
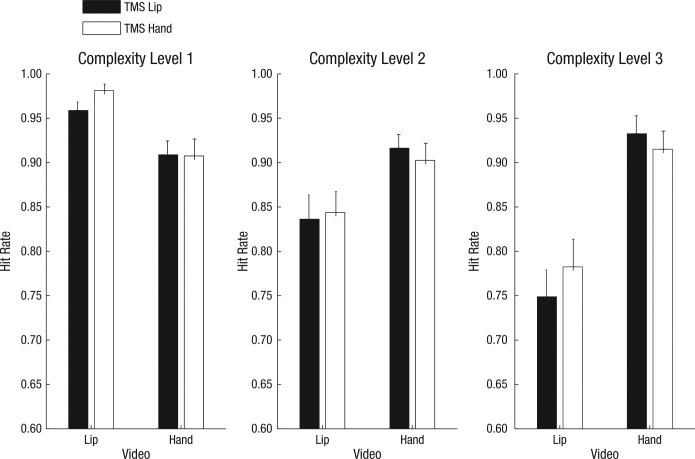
Mean hit rate as a function of the action depicted in the video and the area of premotor cortex over which participants received transcranial magnetic stimulation (TMS). Results are shown separately for each complexity level. Error bars represent standard deviations.

For hit rates (see Fig. 3), a 3 (complexity level: simple, intermediate, complex) × 2 (TMS site: hand, lip) × 2 (video type: hand action, mouth action) analysis of variance (ANOVA) revealed a main effect of complexity level, *F*(2, 38) = 16.58, *p* < .001, η_*p*_^2^ = .47; as complexity level increased, hit rates decreased. A main effect of video type also occurred, *F*(1, 19) = 33.56, *p* < .001, η_*p*_^2^ = .64; specifically, hit rates were lower for mouth actions than for hand actions. There was also a significant interaction between complexity level and video type, *F*(2, 38) = 36.79, *p* < .001, η_*p*_^2^ = .67, with hit rates for mouth actions being more dramatically reduced by increasing complexity than those for hand actions. More important, there was a significant interaction between TMS site and video type, *F*(1, 19) = 6.98, *p* = .016, η_*p*_^2^ = .27. This interaction constituted a double dissociation: cTBS over the hand area specifically impaired participants’ ability to accurately interpret pantomimed hand actions, whereas cTBS over the lip area specifically impaired their ability to accurately interpret mouth actions. No other main effects or interactions reached significance.

For response time (RT), a 3 (complexity level) × 2 (TMS site) × 2 (video type) ANOVA revealed a main effect of complexity level, *F*(2, 38) = 20.82, *p* < .001, η_*p*_^2^ = .52, and a significant interaction between complexity level and video type, *F*(2, 38) = 51.86, *p* < .001, η_*p*_^2^ = .73. There was no two-way interaction between TMS site and video type. No other main effects or interactions reached significance.

## Discussion

The two-way interaction between TMS site (hand vs. lip) and video type (hand action vs. mouth action) for hit rate provides evidence that the premotor hand area plays a causal role in understanding observed hand actions and that the premotor lip area plays a causal role in understanding observed mouth actions. The absence of an interaction of TMS site and video type for RT confirms that the main results (hit rate) were not caused by a trade-off in which participants achieved faster RTs by sacrificing accuracy.

Our results corroborate and extend the findings from several previous studies employing causal methodologies, such as those involving patients with apraxia owing to lesions in PMC and those using TMS to produce virtual lesions. With regard to the former, Pazzaglia et al. (2008) reported that patients with premotor and parietal lesions who were impaired in their ability to perform hand actions were also impaired in their ability to identify sounds typically caused by hand actions, and patients with lesions to premotor areas who were impaired in their ability to move their lips (buccofacial apraxia) were also impaired in their ability to identify sounds typically caused by mouth actions. This is consistent with the findings of Saygin, Wilson, Dronkers, and Bates (2004), who reported that aphasic patients with lesions in left PMC were impaired at a task requiring them to match pictures or names of actions to pictures of the objects used in the actions. Additionally, Moro et al. (2008) reported that patients with lesions in left PMC were specifically impaired at discrimination of bodily actions but not at discrimination of bodily identity.

However, it must be noted that some other lesion studies have yielded contrasting results. For example, Buxbaum, Kyle, and Menon (2005) reported that apraxic patients with parietal lesions were impaired at gesture recognition, whereas apraxic patients with frontal lesions were not. Even more dramatically, Rapcsak, Ochipa, Anderson, and Poizner (1995) conducted a study of G. W., an apraxic patient with bilateral damage to the posterior-superior parietal lobes, who exhibited difficulties in producing pantomimes and in using actual objects but was flawless at recognizing pantomimed actions involving objects.

Because apraxic patients may develop compensatory means of recognizing actions, it is not possible to infer from the negative findings in these latter studies that the mechanisms underlying action performance and action understanding overlap in normal, healthy individuals. It is therefore also important to consider the results of TMS studies. For example, Pobric and Hamilton (2006) demonstrated that virtual lesions created by on-line repetitive TMS (rTMS) over left inferior frontal gyrus (IFG) impaired participants’ ability to estimate the weight of a box lifted by a person but not the weight of a bouncing ball. Also using rTMS to create virtual lesions in left IFG, Tidoni, Borgomaneri, di Pellegrino, and Avenanti (2013) found that participants were impaired in identifying videos of actions in which the actor was attempting to deceive them about the weight of an object, and Urgesi, Candidi, Ionta, and Aglioti (2006) reported that rTMS over left ventral PMC specifically impaired visual discrimination of actions but not visual discrimination of bodily identity. Additionally, using a novel TMS-adaptation paradigm, Cattaneo, Sandrini, and Schwarzbach (2010) found evidence that some neural populations in left ventral PMC encode the goals of perceived actions irrespective of the effector used.

The present experiment also builds on these results in three respects. First, the use of off-line TMS complements earlier studies that used on-line TMS. It must be noted that TMS over frontal areas may induce contraction of facial muscles. Thus, there is a risk of participants being distracted during on-line TMS protocols. Although the aforementioned studies did incorporate matched control tasks or control stimulation sites to minimize the impact of muscular contraction, the present experiment further strengthens their results insofar as it firmly rules out this potential methodological problem.

Second, the present experiment provides evidence that premotor regions contributing to action understanding and action production have a similar somatotopic organization and, thus, that the mechanisms for action understanding and action production overlap. However, it is important to acknowledge that the neural populations in PMC are not likely to be neatly segregated according to the effector that they encode. Indeed, there is evidence of neural populations involved in hand actions in more ventral regions of PMC (Jastorff, Begliomini, Fabbri-Destro, Rizzolatti, & Orban, 2010; Pobric & Hamilton, 2006; Urgesi et al., 2006). Moreover, as noted earlier, Cattaneo et al. (2010) found neural populations in PMC that encode actions in a manner that is not specific to the effector used. Nevertheless, our results strongly suggest that there are also neural populations in left PMC that specialize in encoding observed actions performed by particular effectors.

Third, because the tasks employed here were specifically designed to probe three distinct aspects of action understanding, the results help to adjudicate among competing models of action understanding. Specifically, the results conflict with the inverse-modeling and priming models, which hold that activation of PMC during action observation is subsequent to—and does not play a causal role in—action understanding. Both the direct-matching model and the predictive-coding model, in contrast, predict this pattern of findings and therefore gain support from our results.

Given the absence of any three-way interaction of TMS site, video type, and complexity level (simple, intermediate, complex), our results do not permit any inferences about the hierarchical level at which premotor regions contribute to action understanding (i.e., whether these areas specifically encode low-level kinematics, proximal goals, or distal goals). One possibility is that the areas we targeted encode low-level kinematic information about observed movements and that this kinematic information is relevant for tasks of varying complexity. A follow-up experiment may address this issue by investigating the impact of cTBS on a task in which contextual information is even more crucial than in the complex block of our experiment (e.g., for atypical actions). One might speculate that as contextual information becomes more important and low-level kinematic information less important, the contribution of PMC might decrease and, thus, the impact of cTBS over PMC might diminish.

In sum, our results provide evidence that somatotopically organized regions of PMC contribute causally to action understanding and, thus, that the mechanisms underpinning action understanding overlap with those underpinning action performance. However, these findings do not uniquely support any particular hypothesis about the specific mechanisms by which the neural populations in PMC contribute to action understanding, that is, whether direct matching, predictive coding, somatotopical organization of working memory for observed actions, or some other model best describes the functional mechanism that these neural populations instantiate.
